# A genome-wide association study identifies a susceptibility locus for biliary atresia on 2p16.1 within the gene *EFEMP1*

**DOI:** 10.1371/journal.pgen.1007532

**Published:** 2018-08-13

**Authors:** Ying Chen, Melissa A. Gilbert, Christopher M. Grochowski, Deborah McEldrew, Jessica Llewellyn, Orith Waisbourd-Zinman, Hakon Hakonarson, Joan E. Bailey-Wilson, Pierre Russo, Rebecca G. Wells, Kathleen M. Loomes, Nancy B. Spinner, Marcella Devoto

**Affiliations:** 1 Genomics and Computational Biology Graduate Group, Perelman School of Medicine, University of Pennsylvania, Philadelphia, Pennsylvania, United States of America; 2 Division of Human Genetics, Department of Pediatrics, at The Children's Hospital of Philadelphia, and The Perelman School of Medicine at The University of Pennsylvania, Philadelphia, Pennsylvania, United States of America; 3 Division of Genomic Diagnostics, Department of Pathology and Laboratory Medicine, The Children’s Hospital of Philadelphia, and The Perelman School of Medicine at The University of Pennsylvania, Philadelphia, Pennsylvania, United States of America; 4 Department of Medicine, Perelman School of Medicine, University of Pennsylvania, Philadelphia, Pennsylvania, United States of America; 5 Division of Gastroenterology, Hepatology, and Nutrition, Department of Pediatrics at The Children's Hospital of Philadelphia, and The Perelman School of Medicine at The University of Pennsylvania, Philadelphia, Pennsylvania, United States of America; 6 Schneider Children's Medical Center of Israel, Sackler Faculty of Medicine, Tel-Aviv University, Tel-Aviv, Israel; 7 Center for Applied Genomics, Children's Hospital of Philadelphia, Philadelphia, Pennsylvania, United States of America; 8 Computational and Statistical Genomics Branch, National Human Genome Research Institute, National Institutes of Health, Baltimore, Maryland, United States of America; 9 Division of Anatomic Pathology, Department of Pathology and Laboratory Medicine, Children’s Hospital of Philadelphia and Perelman School of Medicine, University of Pennsylvania, Philadelphia, Pennsylvania, United States of America; 10 Department of Biostatistics, Epidemiology and Informatics, Perelman School of Medicine, University of Pennsylvania, Philadelphia, Pennsylvania, United States of America; 11 Department of Molecular Medicine, Sapienza University, Rome, Italy; Stanford University School of Medicine, UNITED STATES

## Abstract

Biliary atresia (BA) is a rare pediatric cholangiopathy characterized by fibrosclerosing obliteration of the extrahepatic bile ducts, leading to cholestasis, fibrosis, cirrhosis, and eventual liver failure. The etiology of BA remains unknown, although environmental, inflammatory, infectious, and genetic risk factors have been proposed. We performed a genome-wide association study (GWAS) in a European-American cohort of 343 isolated BA patients and 1716 controls to identify genetic loci associated with BA. A second GWAS was performed in an independent European-American cohort of 156 patients with BA and other extrahepatic anomalies and 212 controls to confirm the identified candidate BA-associated SNPs. Meta-analysis revealed three genome-wide significant BA-associated SNPs on 2p16.1 (rs10865291, rs6761893, and rs727878; *P* < 5 ×10^−8^), located within the fifth intron of the *EFEMP1* gene, which encodes a secreted extracellular protein implicated in extracellular matrix remodeling, cell proliferation, and organogenesis. RNA expression analysis showed an increase in *EFEMP1* transcripts from human liver specimens isolated from patients with either BA or other cholestatic diseases when compared to normal control liver samples. Immunohistochemistry demonstrated that EFEMP1 is expressed in cholangiocytes and vascular smooth muscle cells in liver specimens from patients with BA and other cholestatic diseases, but it is absent from cholangiocytes in normal control liver samples. *Efemp1* transcripts had higher expression in cholangiocytes and portal fibroblasts as compared with other cell types in normal rat liver. The identification of a novel BA-associated locus, and implication of *EFEMP1* as a new BA candidate susceptibility gene, could provide new insights to understanding the mechanisms underlying this severe pediatric disorder.

## Author summary

The etiology of biliary atresia (BA) is unknown and likely complex. Environmental, infectious, and genetic risk factors have all been proposed, and the leading hypothesis in the field is that a combination of these factors is responsible for disease manifestation. To identify susceptibility loci for BA, we performed a genome wide association study on two groups of BA patients (one composed of patients with isolated BA (n = 343) and one of patients with BA and other extrahepatic anomalies (n = 156)) and genetically matched controls. We detected a set of SNPs within the *EFEMP1* gene associated with BA in both cohorts. We further showed by immunohistochemistry that EFEMP1 protein was expressed in cholangiocytes and vascular smooth muscle cells in BA livers, and that *EFEMP1* RNA expression levels were elevated in both BA and other cholestatic disease livers. These findings suggest that *EFEMP1* should be considered as a new candidate susceptibility gene for BA.

## Introduction

Biliary atresia (BA; OMIM 210500) is a progressive, necro-inflammatory disease, affecting the extra- and intrahepatic biliary system, leading to bile flow obstruction, cholestasis, and jaundice in infants [1–3]. If left untreated, the condition progresses to hepatic fibrosis, cirrhosis, liver failure, and eventual death within the first two years of life [4]. BA is a rare disease with varying incidence from approximately 1/18,000 live births in Western Europe to 1/8,000 in Asia [5–8]. About 85% of BA patients have a non-syndromic form, in which BA is an isolated finding, while roughly 15% present with other congenital anomalies, including laterality defects in some patients [2, 9]. The primary treatment for BA includes surgical restoration of bile flow (the Kasai hepatoportoenterostomy), which is only successful in about 50% of patients [10]. With or without successful bile drainage, most patients have progressive liver disease. Fifty percent of patients require a liver transplant by age 2 and most of the remaining before reaching adulthood [4, 11]. BA is the most frequent indication for liver transplantation in children worldwide [12].

Although the etiology of BA is not well understood, it is proposed to be multi-factorial and heterogeneous. The current theory posits that BA arises from a combination of genetic predisposition, cholangiocyte damage from environmental factors such as a toxin exposure [13] or viral infection [14], and an inflammatory response to damage [15, 16]. A genetic susceptibility for BA is supported by reports of familial cases, including both parent to child transmission and affected siblings [17–20], and disparate incidences among populations, even after controlling for environmental influences in regional epidemiologic studies [6, 21].

In order to identify genes implicated in susceptibility to BA, candidate gene association studies have been performed, mainly on genes involved in immune or inflammatory responses, and suggestive associations have been found with the genes *ITGB2*, *ADIPOQ*, *IFNG*, *VEGFA*, *MIF* [22–27]. Additional studies, including genome-wide copy number variant (CNV) and single nucleotide polymorphism (SNP) association studies, have identified a few other candidate susceptibility genes. The *GPC1* gene was implicated in BA following a genome-wide CNV association study and its role was further supported by functional studies showing that *gpc1* knockdown in zebrafish led to impaired development of the biliary network [28]. A susceptibility locus on 10q25 near the *XPNPEP1* and *ADD3* genes was identified by a genome-wide SNP association study conducted in Han Chinese BA patients and controls [29], and was replicated in an independent Chinese cohort [30] and in a Thai cohort [31]. Fine-mapping of this signal in North-American patients of European descent [32] and functional studies in an animal model [33] suggested that *ADD3* was the most likely BA susceptibility gene at this locus. A second genome-wide association study (GWAS) performed in a Caucasian cohort identified a signal in the 3' enhancer of *ARF6*, and subsequent studies in zebrafish suggested that knockdown of this gene resulted in biliary defects [34]. Together, these studies have exposed a spectrum of genetic associations in BA and highlight a complex, heterogeneous genetic susceptibility as an emerging feature of the disease etiology.

In an effort to add to our understanding of the genetic susceptibility to BA, we performed a GWAS on 343 patients with isolated BA and 1716 controls of European descent. This analysis identified a novel signal on 2p16.1, in the intronic region of the gene *EFEMP1*, that showed the highest association with BA and reached genome-wide significance after meta-analysis with data from a second cohort of 156 patients with BA and other extrahepatic anomalies and 212 controls, also of European descent. Downstream expression analysis of *EFEMP1* RNA and protein localization in liver specimens suggest that *EFEMP1* may have functional relevance not only in BA, but also in other cholestatic liver diseases.

## Results

### GWAS identifies a candidate BA susceptibility locus on 2p16.1

**Fig 1:**
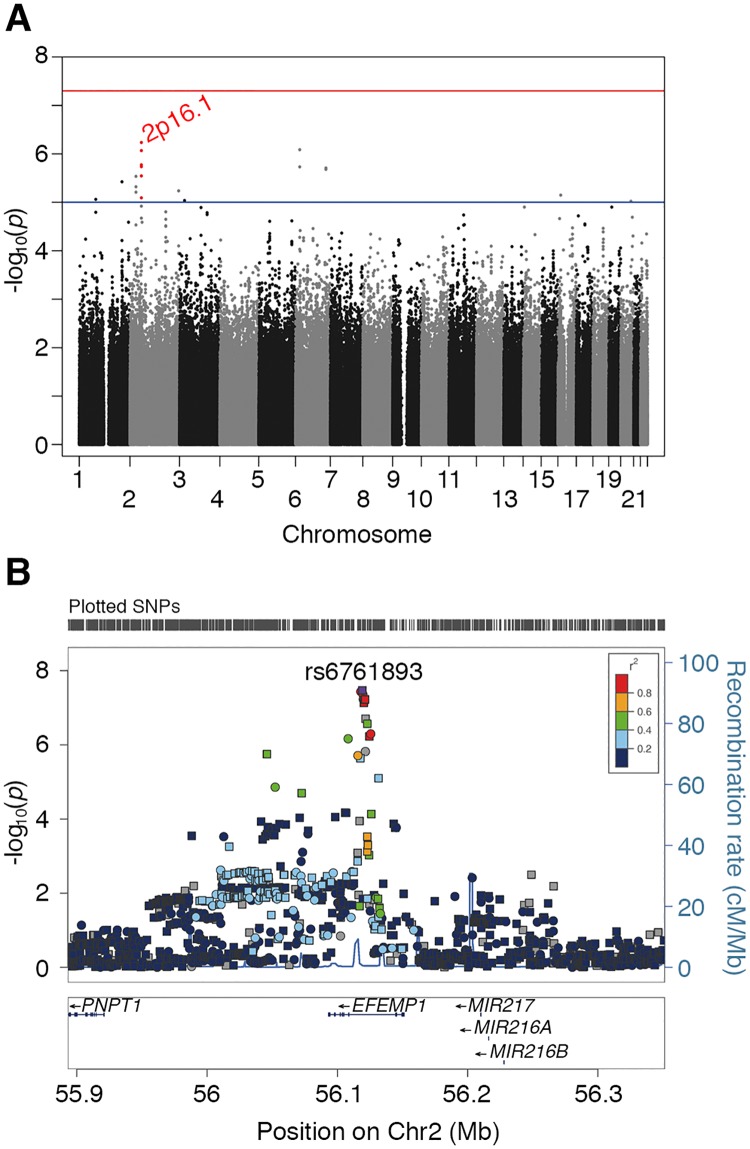
SNPs associated with susceptibility to BA. (A) Manhattan plot of GWAS results in the isolated BA cohort. X-axis: genomic coordinates of tested SNPs. Y-axis: significance level on a -log_10_ scale. The genome-level significance threshold is indicated by the red horizontal line (*P* = 5 × 10^−8^) and the suggestive significance threshold is indicated by the blue horizontal line (*P* = 1 × 10^−5^). (B) Regional association plot of 2p16.1 after meta-analysis. *P*-values (left Y-axis) obtained from additive frequentist association test on genotyped and imputed SNPs. The recombination rate (right Y-axis) is calculated from the 1000 Genomes Phase 3 European ancestry dataset. The top BA-associated SNP is imputed SNP rs6761893 (purple circle), located in the fifth intron of the gene *EFEMP1*. The colors refer to r-square correlation of each SNP to rs6761893 based on the 1000 Genomes Phase 3 European ancestry dataset. The circles represent the genotyped markers and squares represent imputed markers.

We carried out a GWAS in a cohort of 343 isolated BA patients ascertained through the NIDDK-funded Childhood Liver Disease Research Network (ChiLDReN) and 1716 genetically-matched controls of European descent on 1,171,073 common markers (minor allele frequency (MAF) ≥ 5%). Although none of the tested markers reached standard genome-wide significance (*P* < 5 × 10^−8^), 19 markers reached suggestive significance (*P* < 1 × 10^−5^). These 19 markers were located in genomic regions 1p31.1, 1q32.2, 2p23.2, 2p16.1, 2q37.3, 3p24.2, 6p22.3, 6q24.3, 16p13.1, and 20q13.2 (Fig 1A and S1 Table).

The most significant BA-associated SNP was rs10865291, located in the fifth intron of the *EFEMP1* gene on 2p16.1 (*P* = 5.85 ×10^−7^; OR = 1.56) (S1 Table and S1A Fig). The minor allele A was overrepresented in cases (43%) compared to controls (33%). Five neighboring markers in linkage disequilibrium (LD) with rs10865291 (r^2^ > 0.5) also reached suggestive significance (S1 and S2 Tables), indicating that this association signal was unlikely to be due to genotyping errors. After imputation, seven additional SNPs in the same region showed suggestive significance, although the originally identified SNP, rs10865291, remained the most significant (S2A Fig). Association tests conditional on rs10865291 decreased significance for all genotyped and imputed markers within and downstream of the *EFEMP1* gene (S2 Table, S1B and S2B Figs), suggesting that these SNPs contribute to the same association signal.

As a complementary approach to the single SNP association test, we performed a genome-wide gene-based association test using VEGAS2 [35]. The results showed that *EFEMP1* was the top BA-associated gene with empirical *P*-value estimated as 3.9 × 10^−5^ (S3 Table).

We previously replicated, in a smaller subset of the same cases and a different set of controls, the association of BA with SNPs from the 10q25 region identified in a Chinese cohort [29, 32]. To confirm this finding in the current, larger cohort, we imputed the genotypes at rs17095355, the most significant BA-associated SNP identified in the Chinese GWAS, and neighboring markers, since rs17095355 was not present on the Illumina genotyping array used in this study. A few markers, including rs17095355, reached nominal significance (*P* < 0.05). The most significant SNP at the 10q25 locus was rs59804002 (*P* = 0.003), located in the gene *ADD3-AS1*, which encodes a long non-coding RNA (ncRNA) (S3 Fig). In contrast, we were not able to replicate the association of *ARF6* SNPs with BA in our dataset (rs3126184 *P* = 0.85, and rs10140366 *P* = 0.82 after imputation) [34].

To confirm the association with BA of the 2p16.1 SNPs, we performed a second GWAS in an independent group of 156 patients with BA and other extrahepatic anomalies and 212 genetically-matched controls of European descent. All subjects in this cohort were genotyped with the Illumina OmniExpress array, which has a lower SNP density than the Illumina Omni2.5 array used in the isolated BA cohort. Therefore, in order to combine results from the two datasets, we performed genome-wide imputation using 1000 Genomes Project Phase 3 data as the reference panel followed by meta-analysis. The results showed that all SNPs in the 2p16.1 region reaching the threshold for suggestive significance in the isolated BA cohort were more significant following meta-analysis. Importantly, three highly correlated markers (rs10865291, rs6761893, and rs727878; r^2^>0.8) reached genome-wide significance, with imputed SNP rs6761893 showing the highest significance (*P* = 3.39 ×10^−8^) (Table 1 and Fig 1B). No other genomic region, including the other nine regions that had reached suggestive significance in the isolated BA cohort GWAS, contained SNPs that reached genome-wide significance (S4 Fig and S4 Table).

**Table 1:** Meta-analysis on genotyped and imputed SNPs at 2p16.1 reaching P < 1 × 10^−5^ in the isolated BA cohort.

SNP (Position)	Alleles (m/M)*	Isolated BA cohort				Non-Isolated BA cohort				Meta-analysis *P*-value
		MAF (cases)	MAF (controls)	Odds-ratio (95% CI)	*P*-value	MAF (cases)	MAF (controls)	Odds-ratio (95% CI)	*P*-value	
*rs147876856* (56045915)	G/A	0.36	0.27	1.55 (1.30,1.84)	1.81 × 10^−6^	0.32	0.27	1.26 (0.92,1.74)	0.200	1.79 × 10^−6^
rs1346786 (56108333)	T/C	0.39	0.29	1.57 (1.32,1.86)	7.02 × 10^−7^	0.35	0.30	1.26 (0.92,1.72)	0.176	6.86 × 10^−7^
rs11125609 (56115834)	C/T	0.36	0.28	1.51 (1.27,1.80)	6.85 × 10^−6^	0.31	0.29	1.31 (0.95,1.82)	0.093	1.95× 10^−6^
**rs10865291** **(56118046)**	**A/G**	**0.43**	**0.33**	**1.55** **(1.32,1.84)**	**5.55 × 10**^**−7**^	**0.40**	**0.32**	**1.45** **(1.07,1.96)**	**0.020**	**3.66 × 10**^**−8**^
***rs6761893*** **(56119105)**	**T/A**	**0.44**	**0.34**	**1.54** **(1.30,1.81)**	**1.43× 10**^**−6**^	**0.42**	**0.33**	**1.51** **(1.11,2.04)**	**0.007**	**3.39 × 10**^**−8**^
**rs727878** **(56119657)**	**T/C**	**0.43**	**0.33**	**1.55** **(1.32,1.84)**	**6.33 × 10**^**−7**^	**0.40**	**0.32**	**1.45** **(1.07,1.96)**	**0.021**	**4.32 × 10**^**−8**^
rs2868431 (56119967)	A/G	0.44	0.34	1.52 (1.29,1.80)	2.43× 10^−6^	0.42	0.32	1.50 (1.11,2.03)	0.007	5.96 × 10^−8^
*rs6708689* (56120237)	C/A	0.43	0.33	1.54 (1.30,1.82)	1.15× 10^−6^	0.40	0.32	1.45 (1.07,1.97)	0.020	7.40 × 10^−8^
*rs1430193* (56120853)	T/A	0.44	0.34	1.52 (1.29,1.79)	2.58× 10^−6^	0.42	0.32	1.50 (1.11,2.04)	0.007	6.11 × 10^−8^
*rs1430194* (56121320)	G/A	0.44	0.34	1.52 (1.29,1.79)	2.61× 10^−6^	0.42	0.32	1.51 (1.11,2.04)	0.007	6.00 × 10^−8^
*rs3838527* (56121568)	T/TG	0.42	0.32	1.53 (1.29,1.81)	1.65× 10^−6^	0.39	0.31	1.40 (1.03,1.91)	0.037	1.98 × 10^−7^
rs80303336 (56121569)	T/G	0.42	0.32	1.54 (1.30,1.82)	1.52× 10^−6^	NA	NA	NA	NA	1.52× 10^−6^
*rs11887882* (56122892)	G/T	0.50	0.41	1.48 (1.25,1.74)	1.69× 10^−6^	0.45	0.38	1.34 (1.10,1.80)	0.050	2.72 × 10^−7^

Imputed SNPs are italicized. SNPs reaching genome-wide significance are in bold.

*m: minor allele; M: major allele

MAF: minor allele frequency

NA: not available (rs80303336 is not present in the 1000 Genomes Project Phase 3 reference panel)

### eQTL analysis of BA-associated *EFEMP1* SNPs in liver tissue and public databases

To investigate whether the associated SNPs on 2p16.1 were expression quantitative trait loci (eQTLs) with a potential functional role in regulating *EFEMP1* expression in liver tissues, we tested the correlation between the six genotyped SNPs with *P* < 1x10^-5^ (Table 1) and *EFEMP1* expression, using data from a published liver transcriptome study of BA [36]. Genotyping and expression data from 20 BA patients included in both the liver transcriptome study and our GWAS were combined to examine whether the BA-associated SNP genotypes were correlated with *EFEMP1* expression. No correlation was found between the genotypes of any of the six SNPs and *EFEMP1* expression. The results for the two top BA-associated genotyped SNPs (rs10865291 and rs727878) are shown in S5 Fig. Inability to detect correlation may be due to the small sample size (n = 20) or the advanced liver disease status of the BA patients, which may have masked BA-specific effects.

We therefore sought to analyze data from the Genotype-Tissue Expression (GTEx) database V7 (gtexportal.org/), which contains eQTL data from a variety of tissue types from 620 healthy human donors. The level of *EFEMP1* expression was very low in GTEx human liver tissue and we did not detect any liver eQTLs for *EFEMP1* among the BA-associated SNPs within 2p16.1. However, we found that *EFEMP1* expression in GTEx human thyroid tissue was positively correlated with the risk alleles of one of the 2p16.1 BA-associated SNPs, and that this correlation was highly significant (rs1346786, GTEx eQTL *P* = 3.6 × 10^−8^).

### *EFEMP1* transcript levels are differentially-expressed in BA and cholestatic disease liver samples

**Fig 2:**
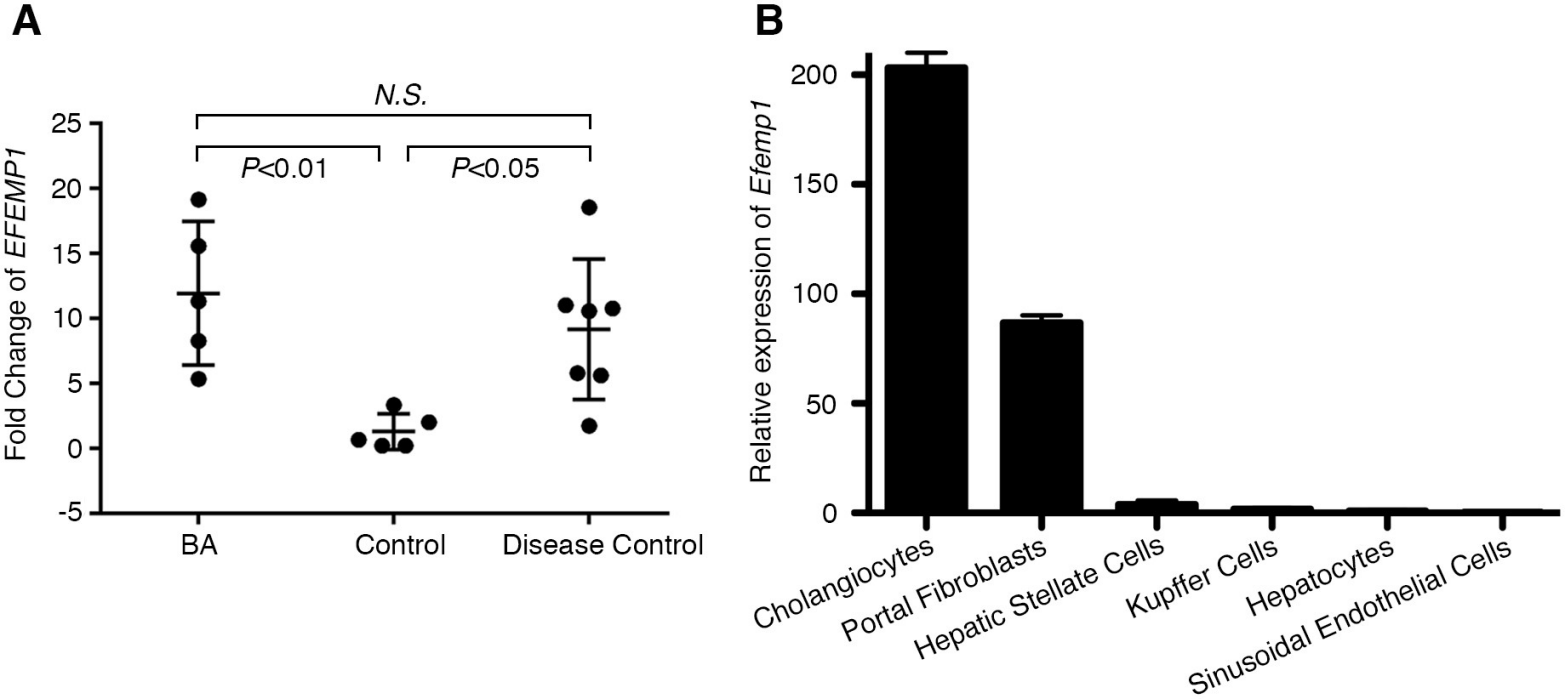
*EFEMP1* gene expression in human and rat liver. (A) ddPCR showing relative expression of *EFEMP1* transcripts in human liver specimens. Fold change of *EFEMP1* was determined by normalizing to the reference gene, *TBP*. Error bars indicate standard deviation. (B) Relative expression of *Efemp1* transcripts in six cell populations from normal rat livers. Rat gene *Rsp12* was used as reference gene. Error bars indicate standard deviation of technical triplicates (cholangiocytes, portal fibroblasts, hepatocytes, Kupffer cells, and sinusoidal endothelial cells) or technical duplicates (hepatic stellate cells).

We next examined the gene expression level of *EFEMP1* in snap-frozen liver samples collected at the time of liver transplant or surgery from patients with BA (n = 5), other cholestatic diseases (n = 7), and non-cholestatic controls (n = 5) using droplet digital PCR (ddPCR) (S5 Table). We found that *EFEMP1* expression was significantly different among the three groups (one-way ANOVA *P* = 0.007), being increased in both BA livers and other cholestatic disease livers compared to non-cholestatic control livers (Fig 2A). This finding is consistent with a previous observation that *EFEMP1* expression is upregulated in BA patients by 2.85 fold when compared to controls without cholestatic liver diseases [36]. We observed variable gene expression levels among BA patient samples, and there seemed to be a correlation between age at transplant and transcript expression levels (S6 Fig). Patients who received a transplant at an earlier age showed the highest levels of *EFEMP1* RNA expression, while patients who received a transplant at an older age showed the lowest levels of RNA expression.

### EFEMP1 is expressed in cholangiocytes and portal fibroblasts in rat liver and is upregulated in bile ducts in BA and other cholestatic diseases

To examine the cell type-specific expression pattern of *EFEMP1*, we quantified the orthologous *Efemp1* transcripts in six rat liver cell populations using ddPCR. In normal rats, we found that *Efemp1* is significantly enriched in cholangiocytes and portal fibroblasts compared to hepatocytes, Kupffer cells, sinusoidal endothelial cells, and hepatic stellate cells (one-way ANOVA *P*<0.0001) (Fig 2B).

**Fig 3:**
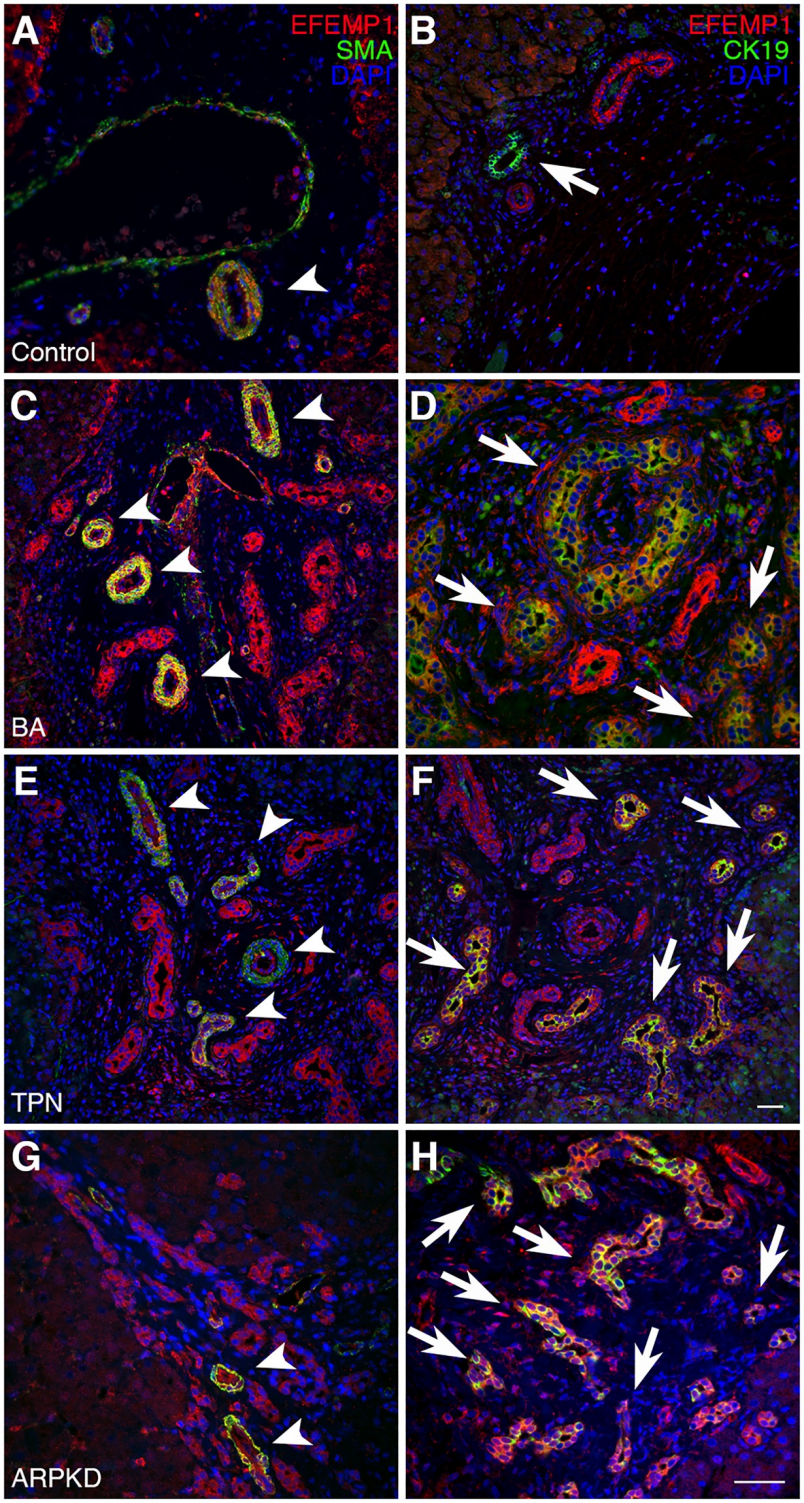
Localization of EFEMP1 in control and cholestatic disease human liver samples. (A) EFEMP1 is expressed in SMA positive smooth muscle cells, but not (B) CK19 positive intrahepatic cholangiocytes in control liver. EFEMP1 is expressed in both smooth muscle cells and intrahepatic cholangiocytes in (C, D) BA, (E, F) TPN, and (G, H) ARPKD liver. Scale bar = 25 μm. (A-F) were taken at a lower magnification and correspond to the scale bar in (F). (G, H) were taken at a higher magnification and correspond to the scale bar in (H). Arrowheads indicate vascular smooth muscle cells and arrows indicate cholangiocytes.

**Fig 4:**
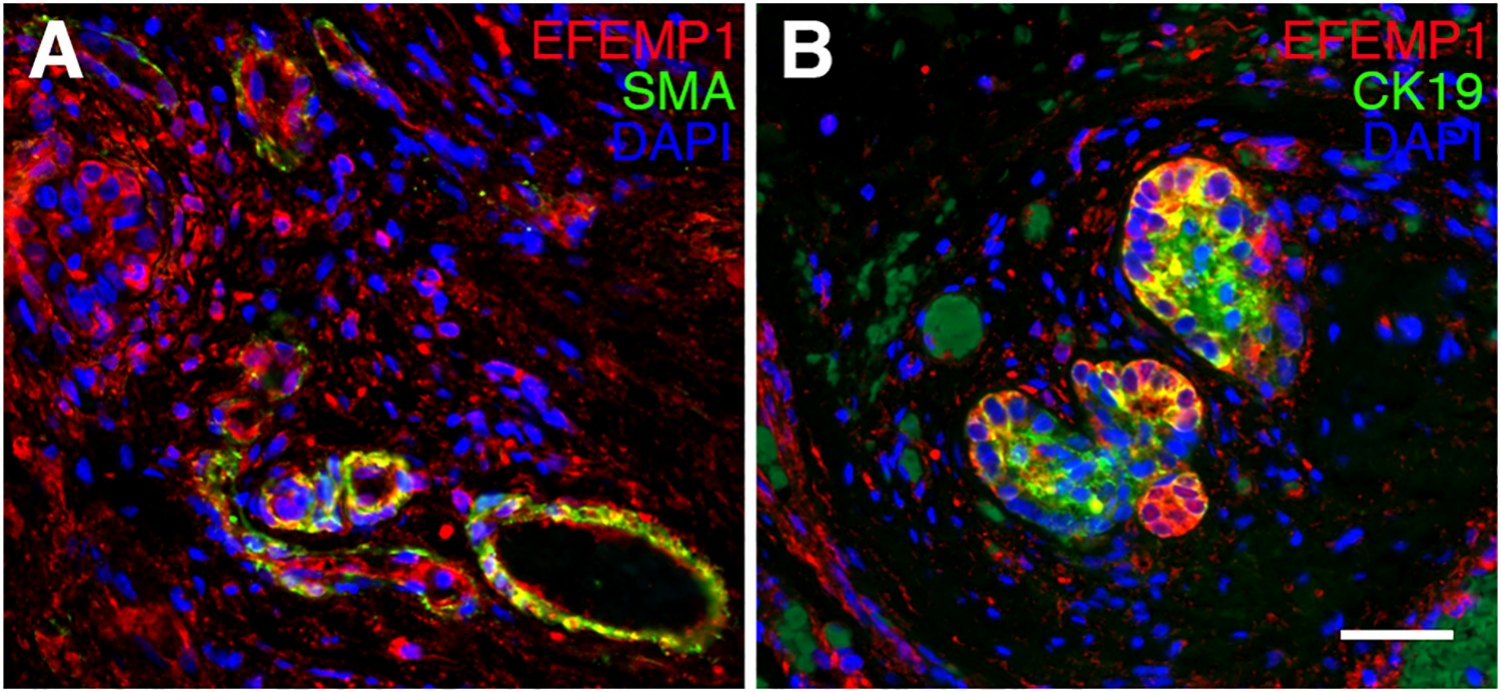
EFEMP1 expression in BA extrahepatic bile ducts. EFEMP1 is expressed in both (A) SMA positive smooth muscle cells and (B) CK19 positive extrahepatic cholangiocytes in BA liver. Scale bar = 25 μm.

To investigate the expression and localization of EFEMP1 protein in human liver, we performed immunohistochemistry (IHC) on normal control and BA livers, and we also included disease control livers from patients with total parenteral nutrition-associated (TPN) cholestasis and autosomal recessive polycystic kidney disease (ARPKD). We found that EFEMP1 is specifically expressed in α-smooth muscle actin (α-SMA)-positive vascular smooth muscle cells (Fig 3A and S7A and S7B Fig), but not in cytokeratin 19 (CK19)-positive intrahepatic cholangiocytes (Fig 3B and S7C and S7D Fig) in normal control livers. In contrast, it is expressed in both vascular smooth muscle cells and intrahepatic cholangiocytes in BA livers and in livers of patients with TPN cholestasis and ARPKD (Fig 3C–3H and S7E–S7P Fig). Immunohistochemical staining for EFEMP1 in the extrahepatic bile duct remnant removed from a BA patient during Kasai hepatoportoenterostomy showed that it is also expressed in extrahepatic cholangiocytes, as well as in vascular smooth muscle cells (Fig 4 and S8 Fig).

## Discussion

Using a GWAS approach, we identified a novel candidate susceptibility locus for BA that maps within the *EFEMP1* gene on 2p16.1. *EFEMP1* encodes the EGF-containing fibulin-like extracellular matrix protein, Fibulin-3 (EFEMP1), which is one of seven members of the fibulin family and has roles in extracellular matrix remodeling, tissue regeneration, and organogenesis [37, 38]. EFEMP1 has also been reported to activate Notch signaling *in vitro*, although with less efficiency than JAG1 [39], and to exert both tumor suppressive and oncogenic effects in various cancers by participating in multiple signaling pathways [40–43]. A missense mutation in *EFEMP1* (R345W) has been found in patients with Doyne honeycomb retinal dystrophy (DHRD) [44]. Consistent with this finding, *Efemp1*-R345W knock-in mice develop a similar ocular phenotype, supporting the causal role of the *EFEMP1* R345W mutation in DHRD [45]. In contrast, targeted inactivation of *Efemp1* in mice resulted in premature aging, shortened lifespan and reduced reproductive capacity compared to wild-type mice, without signs of macular degeneration [46]. In aged *Efemp1*^*-/-*^ mice (18 to 24 months of age), the authors observed overall decreased body mass, muscle and fat mass compared to littermate controls. In addition, the aged mutant mice had significantly decreased mass of internal organs including liver, spleen and kidney. A biliary phenotype was not reported. Of note, adult *Efemp1*^*-/-*^ mice on the C57BL/6 background developed herniation of abdominal and pelvic organs. On further examination, this phenotype was found to be due to reduction of elastic fibers in the abdominal fascia [46]. Variants in the *EFEMP1* locus have been associated with a variety of human traits and diseases, such as human height [47], forced vital capacity [48], inguinal hernia [49], and chronic venous disease (CVD) [50]. We note that it is not unusual for genes to be associated with both complex and Mendelian disorders affecting different organ systems [51].

Using two different cohorts in two separate GWAS, we were able to identify a region of interest within 2p16.1 in a European-American population of isolated BA patients, and we subsequently observed additional support for this association in an independent European-American cohort of patients with BA and other extrahepatic anomalies. When results from these two cohorts were combined using meta-analysis, three markers (rs10865291, rs6761893, and rs727878) reached genome-wide significance, providing evidence towards disease relevance. The associated alleles at these three SNPs are very common in the East Asian population (rs10865291:A = 0.77; rs6761893:T = 0.88; and rs727878:T = 0.77; 1000 Genomes Project Phase 3 data), which is consistent with a higher documented incidence of BA in Asia [52]. We also confirmed replication in our European American cohort of an association signal within 10q25, originally identified in a GWAS performed in a Chinese cohort [29].

All of the BA-associated SNPs within 2p16.1 identified in our GWAS, including the top three markers (rs10865291, rs6761893, and rs727878), are located in the 3' UTR or within intronic regions of the *EFEMP1* gene. Although we did not detect any correlation between the genotypes of BA-associated SNPs and *EFEMP1* gene expression in livers from BA patients, analysis of data from the GTEx project showed that one of the BA-associated SNP (rs1346786) is an eQTLs for *EFEMP1* in healthy human thyroid tissue. This result suggests that the BA-associated SNPs (or other SNPs in linkage disequilibrium with them) may play a regulatory role in *EFEMP1* expression in specific tissues or at specific time-points. RNA expression data showed that *EFEMP1* was strongly upregulated in BA liver when compared to healthy liver. This result is consistent with a published comparative liver transcriptome analysis, which showed that *EFEMP1* expression was upregulated in BA patients by 2.85 fold in comparison with controls without cholestatic liver diseases [36]. We also found EFEMP1 protein expression in vascular smooth muscle cells in both control and disease liver specimens, an expression profile that is consistent with a published report of EFEMP1 as a candidate susceptibility gene for CVD [50].

The identification of *EFEMP1* as a susceptibility locus in both isolated BA and BA with other anomalies, which are clinically-distinct classifications of BA, suggests that it may be important in liver and bile duct development. Recent evidence has shown that direct bilirubin levels are elevated in the first days of life even in infants with isolated BA, supporting a model of prenatal onset of disease pathogenesis in the absence of other congenital anomalies [53]. Fibulins are known to have extensive interactions with laminins and other extracellular matrix (ECM) -related proteins [38]. Given the close proximity and cellular communication of the developing bile ducts with the ECM of the portal mesenchyme, it is possible that EFEMP1 may have a previously unrecognized function in bile duct development.

We found *Efemp1* transcripts to have higher expression in cholangiocytes and portal fibroblasts as compared with other cell types in normal rat liver. In human whole liver tissue, *EFEMP1* RNA expression was high not only in BA, but also in non-BA cholestatic disease as compared with control liver tissue. It is interesting that expression of *Efemp1* transcripts was high in rat cholangiocytes and low in human control whole liver tissue, although we note that the major cell type in the liver is hepatocytes, which express very low levels of *Efemp1* RNA in the rat (Fig 2). We further investigated cell type-specific expression of EFEMP1 protein in human liver tissue by IHC, which showed EFEMP1 expression in CK19 positive cholangiocytes in both BA and other cholestatic disease patient liver, in contrast to healthy control liver where EFEMP1 protein was not expressed in bile ducts. This distinct expression pattern suggests that EFEMP1 may have a bile duct-specific role in cholestatic liver disease. More specifically, the similar expression pattern of *EFEMP1* in both BA and other cholestatic diseases suggests a common function for EFEMP1 in cholestatic liver disease. Given that progressive fibrosis is a common feature of disease pathogenesis for these various conditions, EFEMP1 may have a role in fibrosis. Indeed, there was advanced fibrosis in all the BA and disease control samples used in this study. This is further supported by known roles for EFEMP1 as an ECM protein, and the importance of the ECM in a variety of biological processes, including fibrosis [37]. Moreover, it has been reported that portal fibroblasts are important sources of matrix proteins in hepatic fibrosis [54]. Our liver cell type-specific expression analysis of *Efemp1* in normal rats showed it was highly expressed in portal fibroblasts compared with other liver cell populations, which also supports a possible role in hepatic fibrosis.

Identification of the causal gene and the underlying causal mechanism following a significant GWAS signal is a challenging task [55]. Given the intronic localization of our genome-wide significant associated SNPs, we focused initially on evaluating a potential role for *EFEMP1* in BA by gene expression and protein localization studies. It is still possible that other genes may be involved, in addition to or in place of *EFEMP1*, through complex long-range interactions or regulatory mechanisms. Other studies have shown that the genes targeted by GWAS-identified SNPs are not always or only the nearest ones [56]. Our data support a role for *EFEMP1* in the etiology of BA, and its possible involvement in other cholestatic liver diseases. However, with the available data it is difficult to speculate on what the underlying mechanism linking the GWAS identified *EFEMP1* common variants to susceptibility to BA might be, and whether their effect could be mediated through a gain or loss of function of the EFEMP1 protein. Significant evidence supports a model of BA resulting from a combination of an environmental insult (such as exposure to toxins or viruses) and host susceptibility to the insult and/or to a damaging and progressive response to it [57]. In this context, it is perhaps not surprising that, in the absence of an environmental insult, the available *Efemp1* mouse models do not show a biliary phenotype [45, 46]. Further studies will be necessary to define the possible role of *EFEMP1* in the development of BA, and in progression of fibrosis in BA or other cholestatic liver diseases. Ultimately, its identification as a candidate BA susceptibility gene could provide new insights to understanding the mechanisms underlying BA pathogenesis.

## Materials and methods

### Subjects and sample collection

BA patient samples were obtained from the National Institute of Diabetes and Digestive and Kidney Diseases (NIDDK)-funded Childhood Liver Disease Research Network (ChiLDReN). Patients were enrolled into the ChiLDReN study at each participating site under IRB-approved protocols, and patients’ data and blood samples had been obtained with informed consent from patients’ parents or legal guardians. DNA samples from participants were isolated and banked at the Rutgers University NIDDK biorepository. De-identified DNA samples were sent to our laboratory for analysis. All patients were diagnosed with BA based on clinical presentation, liver histology, and intraoperative cholangiogram. The majority had their diagnosis confirmed by examination of the biliary remnant from portoenterostomy. A total of 450 self-reported white patients with isolated BA (260 females and 190 males), including 74 Hispanics, and 170 self-reported white, non-Hispanic, patients with BA and other extrahepatic anomalies (104 females and 66 males) were genotyped.

Control genotypes for the isolated BA samples were obtained from de-identified samples of 1981 self-reported white, healthy individuals (1167 females and 814 males) from the NIH-funded Age-Related Eye Disease Study (AREDS) [58]. Controls for the patients with BA and other anomalies were de-identified samples of 246 self-reported white children (187 females and 59 males) genotyped at the Center for Applied Genomics (CAG) at the Children’s Hospital of Philadelphia (CHOP). These children had no diagnosis of congenital anomalies or any diseases in the digestive system.

De-identified human liver specimens used for the gene expression assay were obtained from a CHOP liver repository. All samples were collected at time of liver transplant or surgery, and included non-cholestatic livers (n = 5), cholestatic disease livers (n = 7) and BA livers (n = 5). Non-cholestatic controls included patients with citrullinemia (n = 2) and propionic acidemia (n = 1), and normal liver tissue adjacent to tumor (n = 2). The cholestatic disease control group consisted of children with Alagille syndrome (n = 3), cystic fibrosis (n = 1), primary sclerosing cholangitis (n = 2), and autoimmune hepatitis (n = 1). The characteristics of these 17 human liver specimens are summarized in S5 Table.

### SNP genotyping and quality control

DNA samples from isolated BA patients and AREDS control subjects were genotyped with the Illumina Omni2.5 BeadChip (Illumina, San Diego, CA). Per-sample and per-marker quality control was performed using PLINK [59]. SNPs with genotype missing rate >5%, minor allele frequency (MAF) <5%, missing rate significantly different between cases and controls (*P* <0.00001), or significant deviation from Hardy-Weinberg equilibrium in controls (P <0.00001) were excluded. A total of 1,171,073 markers were included in the association analysis. Samples with a genotype call rate < 97%, discordance between reported and genotyped sex, and an outlying heterozygosity rate (defined as outside of 3 standard deviation from the mean) were excluded. Duplicates and samples with hidden relatedness were identified by estimating identity-by-descent (IBD) on the basis of the genome-wide identity-by-state (IBS) information, and one from each pair of samples with proportion of IBD larger than 0.105 was excluded.

To control for population stratification, principal component analysis (PCA) was carried out in the remaining 432 BA cases and 1876 controls by incorporating the genotypes of HapMap3 individuals from 11 populations (S9 Fig). Genetically-matched cases and controls were selected based on the first two principal components (PCs) using an algorithm called Ordering Points to Identify the Clustering Structure (OPTICS) [60] with the maximum distance in a cluster set as 0.003 (S10 Fig). A total of 343 cases and 1716 controls that clustered with the HapMap3 samples of European ancestry were selected for the association test. A Tracy-Widom test was performed to estimate the number of statistically significant PCs to be included in the association test to correct for any potential residual population stratification.

DNA from 170 patients with BA and other anomalies and 246 CAG controls were genotyped with the Illumina OmniExpress BeadChip. Quality control was applied exactly as described for the isolated BA cohort. A total of 156 BA and 212 genetically-matched controls of European ancestry were selected for association test on 579,213 markers.

### Association tests and imputation

Adjusted logistic regression under the additive model, with the statistically significant PCs as covariates, was carried out for case-control association analysis in the isolated BA cohort for 1,171,073 genotyped common SNPs (MAF ≥ 5%). Genome-wide significance was set at *P* < 5x10^-8^, and suggestive association was defined as an average of 1 false-positive association per GWAS in European populations, or *P* < 1x10^-5^ [61]. No deviation from the expected *P*-values was observed in the Q-Q plot and the genomic inflation factor λ was estimated as 1 (S11 Fig). The *P*-values of each marker within 20kb upstream and downstream of a gene were used for gene-based association analysis in the isolated BA cohort using VEGAS2 [35].

IMPUTE2 [62] v2.3.2 was used for imputation of all SNPs and indel variants annotated in the 1000 Genomes Project Phase 3 in both BA cohorts and their respective controls. Variants that were imputed with low confidence (info < 0.8), or with MAF < 0.05 were removed from subsequent association test. Testing for association with BA under an additive genetic effect model, adjusting for the statistically significant PCs, was performed using the frequentist likelihood score method implemented in SNPTEST [63] v2.5 in the two datasets separately. Meta-analysis across the two cohorts was performed with METAL [64] using the inverse variance method, which weights the effect size estimates (*β*-coefficients) by their estimated standard errors.

Regional association plots were obtained with LocusZoom [65].

### Gene expression assay

Total RNA was extracted from 17 human liver specimens using TRIzol reagent (Invitrogen, Carlsbad, CA) and the RNeasy RNA extraction kit (Qiagen, Venlo, The Netherlands) according to the manufacturer’s instructions. Subsequent cDNA was synthesized using the TaqMan Reverse Transcription Kit under normal conditions (Applied Biosystems, Foster City, CA). ddPCR was performed on a Bio-Rad QX100 ddPCR system (Bio-Rad, Hercules, CA) using standard methods. Droplets were generated from reactions containing 60 ng of cDNA from each of the 17 liver biopsy specimens using the TaqMan fluorescent reporter assay (Thermo Fisher Scientific, Waltham, MA). Human *EFEMP1* primer and probe set labeled with FAM (Hs00244575_m1) and human *TBP* primer and probe set labeled with VIC (Hs00427620_m1) were used. Samples were multiplexed and run under standard ddPCR conditions [66]. One-way analysis of variance was performed to test for a difference among the three groups (non-cholestatic control, cholestatic disease control and BA) and *P* < 0.05 was considered as statistically significant.

Hepatocytes, Kupffer cells, portal fibroblasts, sinusoidal endothelial cells, and hepatic stellate cells were isolated from normal rats [67], and cholangiocytes were isolated as the non-adhering cells after 1 hour of plating, using the same protocol as portal fibroblasts. RNA was extracted from single specimens for each cell type as described [67]. cDNA was generated using the TaqMan Reverse Transcription Kit (Applied Biosystems, Foster City, CA). Droplets for ddPCR were generated from reactions containing 30 ng of cDNA from each of the cell populations using TaqMan (Thermo Fisher Scientific, Waltham, MA) rat *Efemp1* primer and probe set labeled with FAM (Rn01434325_m1) and TaqMan rat *Rps12* primer and probe set labeled with VIC (Rn00821373_g1) as the control. One-way analysis of variance was performed to test for a difference among three cell type groups (cholangiocytes, portal fibroblasts and all others) and *P* < 0.05 was considered as statistically significant.

### Immunohistochemistry

Formalin-fixed, paraffin-embedded liver slides were obtained from the Department of Pathology at CHOP. These slides were taken from de-identified liver samples not required for clinical use. Samples included BA tissue from liver explants, BA tissue from extrahepatic bile ducts (from Kasai hepatoportoenterostomy), control liver tissue from an autopsied infant with no known liver disease, tissue from an infant with TPN, and tissue from an infant with clinically-diagnosed ARPKD.

Tissue was de-paraffinized using standard procedures. All tissue was pre-treated by exposing to 3% hydrogen peroxide in methanol for 5 minutes followed by heat-mediated antigen retrieval in citrate buffer (Sigma-Aldrich, St. Louis, MO) for 10 minutes. Slides were blocked for one hour at room temperature (10% donkey serum, 5% milk, 4% BSA, 0.1% Triton X-100 in PBS) and incubated in primary antibody overnight at 4°C. All primary antibodies were purchased from Abcam (Cambridge, MA), and included CK19 (1:500; ab7754), α-smooth muscle actin (1:50; ab7817), and EFEMP1 (1:100; ab151976). Slides were incubated in secondary antibodies at a dilution of 1:500 for 1 hour at room temperature (Alexa Fluor 488 α-mouse and Alexa Fluor 555 α-rabbit, Life Technologies, Carlsbad, CA). All slides were mounted with Vectashield (Vector Laboratories, Inc., Burlingame, CA) and photographs were taken on a Leica DMi8 inverted microscope using Leica Application Suite (LAS X) software (Wetzlar, Germany).

## Supporting information

S1 Fig Regional association plot of 2p16.1 in the isolated BA cohort before imputation. (A) Six markers in this region had *P*-value < 1x10^-5^, including rs10865291, which showed the strongest association. (B) Association test results conditional on rs10865291. *P*-values calculated from adjusted logistic regression test under additive genetic model.
(TIF)

S2 Fig Regional association plot of 2p16.1 in the isolated BA cohort after imputation. (A) Association test results after imputation with the 1000 Genomes Project Phase 3 data. SNP rs10865291 remained the most significant one. (B) Association test results after imputation with the 1000 Genomes Project Phase 3 data conditional on rs10865291. *P*-values generated under an additive genetic effect model using the frequentist likelihood score method implemented in SNPTEST v.2.5.
(TIF)

S3 Fig Regional association plot of 10q25 in the isolated BA cohort after imputation. Association test results after imputation with the 1000 Genomes Project Phase 3 data. *P*-values generated under an additive genetic effect model using the frequentist likelihood score method implemented in SNPTEST v.2.5. The most highly associated SNP in this region was rs59804002 (*P*-value = 0.003). The association signal is located in the gene *ADD3-AS1* encoding a long ncRNA.
(TIF)

S4 Fig Association results after meta-analysis on two BA cohorts. (A) Manhattan plot of association results after meta-analysis on two BA cohorts. (B) Q-Q plot of association results after meta-analysis on two BA cohorts. The observed *P*-values from adjusted logistic regression are plotted against the expected *P*-values assuming a null hypothesis of no association.
(TIF)

S5 Fig Correlation of genotypes at the two top BA-associated genotyped SNPs and relative *EFEMP1* gene expression in explant livers from 20 BA patients. Relative *EFEMP1* gene expression was obtained from a published liver comparative transcriptome study (36). (A) The genotypes of SNP rs727878 (T/C) are plotted on the x-axis. T is the risk allele. (B) The genotypes of SNP rs10865291 (A/G) are plotted on the x-axis. A is the risk allele.
(TIF)

S6 Fig Age at transplant and relative *EFEMP1* RNA expression. Five human liver samples were obtained during surgery from BA patients who had liver transplants at various ages (0.74, 0.81, 1.07, 5.47, and 11.17 years). Relative expression of *EFEMP1* RNA is plotted for each individual sample on the y-axis against the age at transplant on the x-axis.
(TIF)

S7 Fig Split channels for all staining shown in Fig 3. SMA (A1) and EFEMP1 (A2) staining in control tissue; CK19 (B1) and EFEMP1 (B2) staining in control tissue. SMA (C1) and EFEMP1 (C2) staining in BA tissue; CK19 (D1) and EFEMP1 (D2) staining in BA tissue. SMA (E1) and EFEMP1 (E2) staining in TPN tissue; CK19 (F1) and EFEMP1 (F2) staining in TPN tissue. SMA (G1) and EFEMP1 (G2) staining in ARPKD tissue; CK19 (H1) and EFEMP1 (H2) staining in ARPKD tissue.
(TIF)

S8 Fig Split channels for staining of extrahepatic bile duct shown in Fig 4. SMA (A1) and EFEMP1 (A2); CK19 (B1) and EFEMP1 (B2) staining in BA tissue.
(TIF)

S9 Fig Plot of the first two PCs of 432 isolated BA cases and 1876 AREDS controls with HapMap3 samples (11 populations). Cases are shown in red and controls are shown in black.
(TIF)

S10 Fig Selection of ancestry-matched isolated BA cases and controls. Cases are shown in red and controls are shown in black. The OPTICS clustering algorithm was applied to the first two PCs of 432 BA cases and 1876 AREDS controls. Green circles the selected 343 cases and 1716 controls of European ancestry, which are in one cluster with distance less than 0.003.
(TIF)

S11 Fig Q-Q plot of association results in the isolated BA cohort. The observed *P*-values from adjusted logistic regression are plotted against the expected *P*-values assuming a null hypothesis of no association. No evidence of inflation due to population stratification or other sources of bias was detected. Genomic inflation factor (λ) was estimated as 1.
(TIF)

S1 Table Genotyped SNPs reaching the suggestive significance threshold (*P* < 1x10^-5^) in the isolated BA cohort. (DOCX)

S2 Table Conditional association test on genotyped SNPs in the *EFEMP1* gene that reached suggestive significance in the isolated BA cohort. (DOCX)

S3 Table List of top 25 genes in gene-based association analysis in the isolated BA cohort. (DOCX)

S4 Table Meta-analysis on 13 genotyped SNPs outside of 2p16.1 reaching *P* < 1 × 10^−5^ in the isolated BA cohort. (DOCX)

S5 Table Characteristics of 17 human liver specimens used for *EFEMP1* expression analysis. (DOCX)
