# Supplementary figures and images for: A genome-wide association study identifies a susceptibility locus for biliary atresia on 2p16.1 within the gene EFEMP1
Source: PLoS Genet. 2018 Aug 13;14(8):e1007532. doi: 10.1371/journal.pgen.1007532 (PMC6107291; doi:10.1371/journal.pgen.1007532)

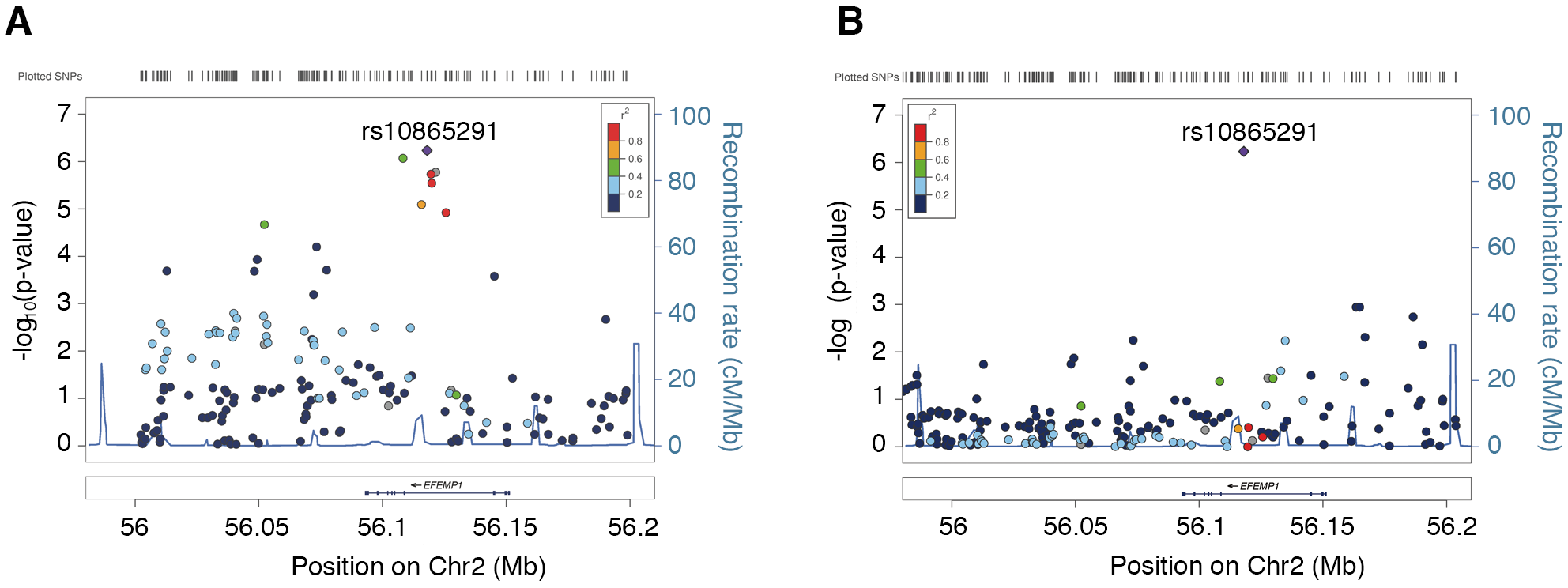

Supplement: S1 Fig — (A) Six markers in this region had P-value < 1x10^-5^, including rs10865291, which showed the strongest association. (B) Association test results conditional on rs10865291. P-values calculated from adjusted logistic regression test under additive genetic model. (TIF) [file pgen.1007532.s001.tif]

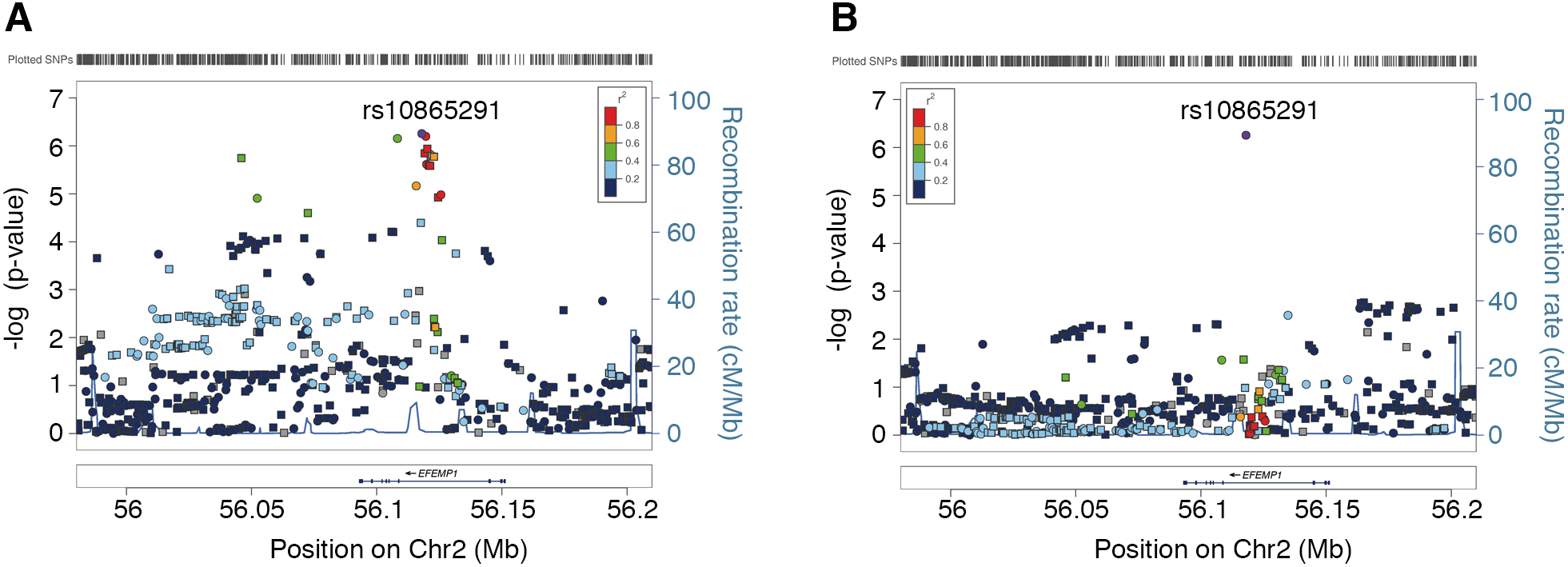

Supplement: S2 Fig — (A) Association test results after imputation with the 1000 Genomes Project Phase 3 data. SNP rs10865291 remained the most significant one. (B) Association test results after imputation with the 1000 Genomes Project Phase 3 data conditional on rs10865291. P-values generated under an additive genetic effect model using the frequentist likelihood score method implemented in SNPTEST v.2.5. (TIF) [file pgen.1007532.s002.tif]

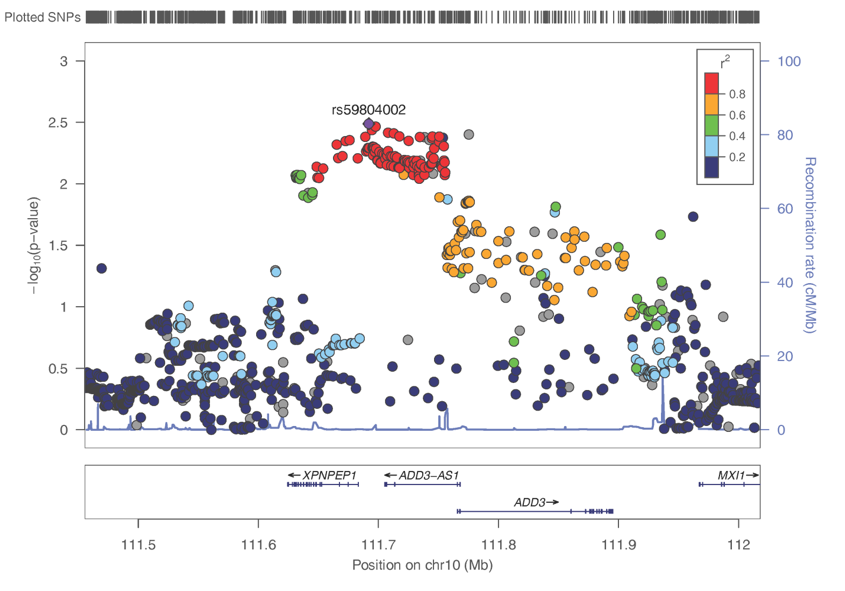

Supplement: S3 Fig — Association test results after imputation with the 1000 Genomes Project Phase 3 data. P-values generated under an additive genetic effect model using the frequentist likelihood score method implemented in SNPTEST v.2.5. The most highly associated SNP in this region was rs59804002 (P-value = 0.003). The association signal is located in the gene ADD3-AS1 encoding a long ncRNA. (TIF) [file pgen.1007532.s003.tif]

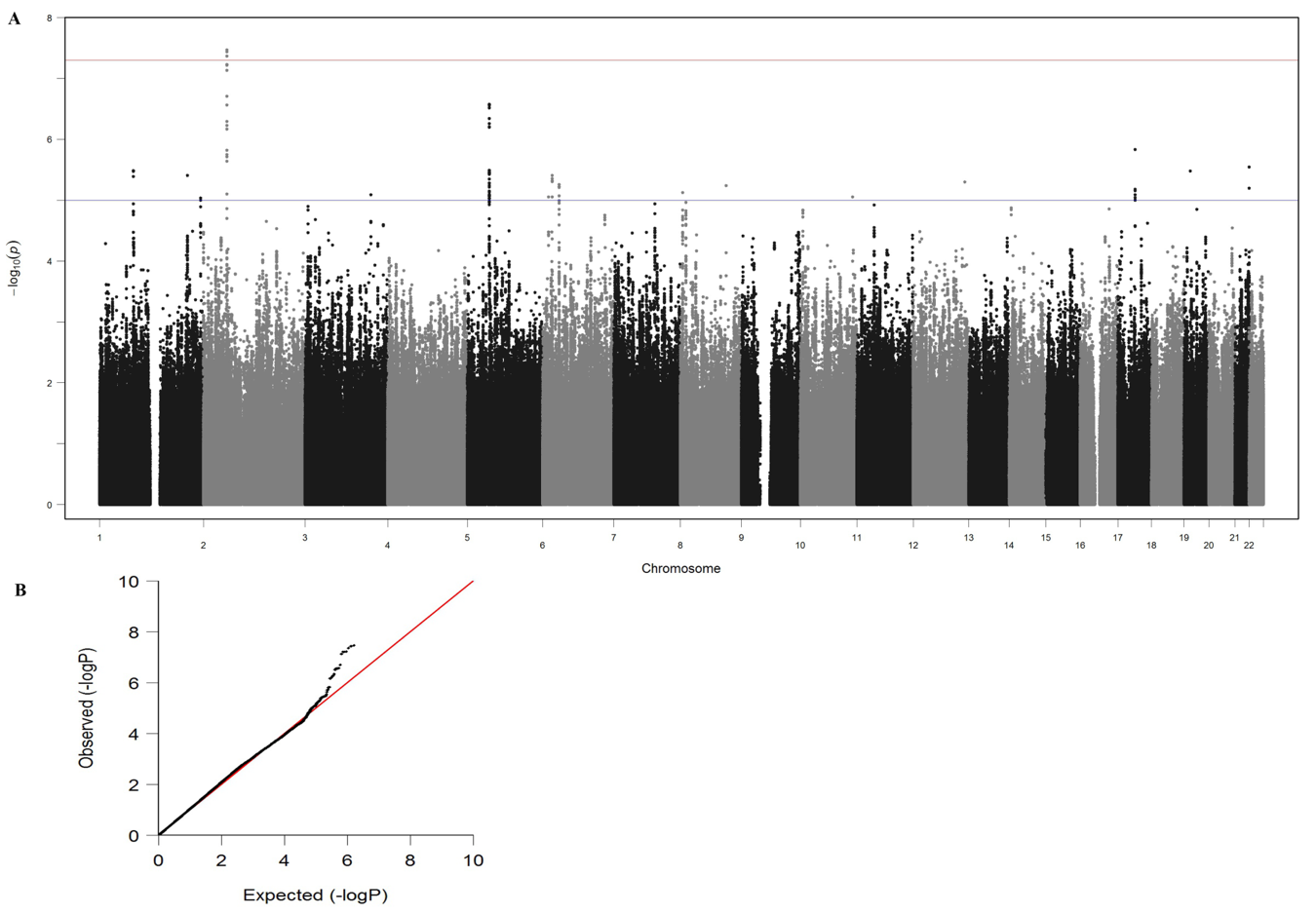

Supplement: S4 Fig — (A) Manhattan plot of association results after meta-analysis on two BA cohorts. (B) Q-Q plot of association results after meta-analysis on two BA cohorts. The observed P-values from adjusted logistic regression are plotted against the expected P-values assuming a null hypothesis of no association. (TIF) [file pgen.1007532.s004.tif]

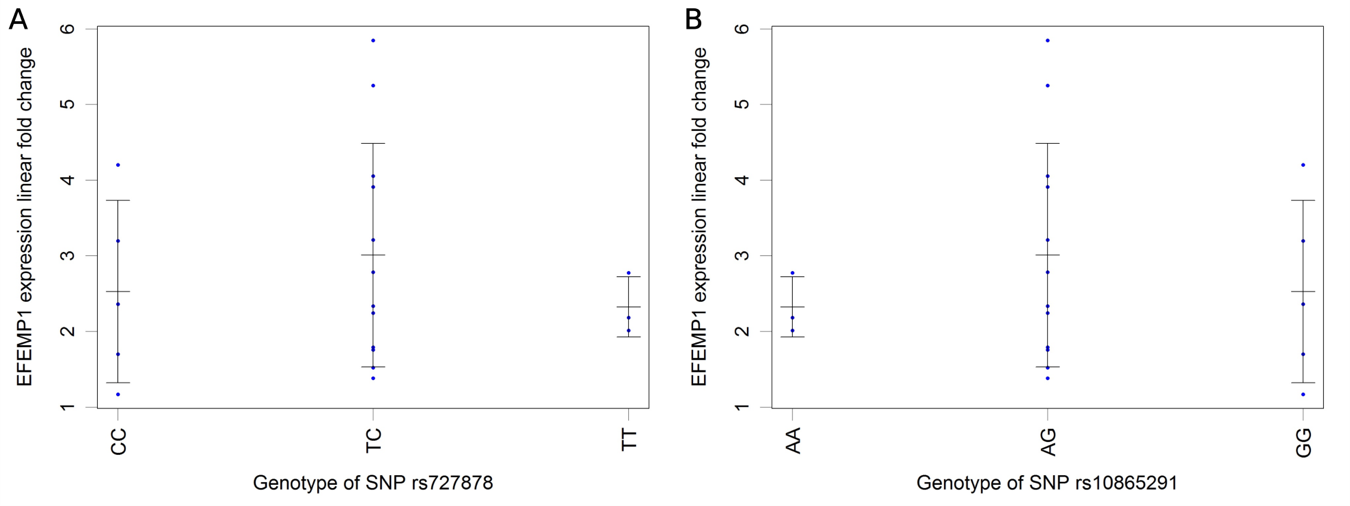

Supplement: S5 Fig — Relative EFEMP1 gene expression was obtained from a published liver comparative transcriptome study (36). (A) The genotypes of SNP rs727878 (T/C) are plotted on the x-axis. T is the risk allele. (B) The genotypes of SNP rs10865291 (A/G) are plotted on the x-axis. A is the risk allele. (TIF) [file pgen.1007532.s005.tif]

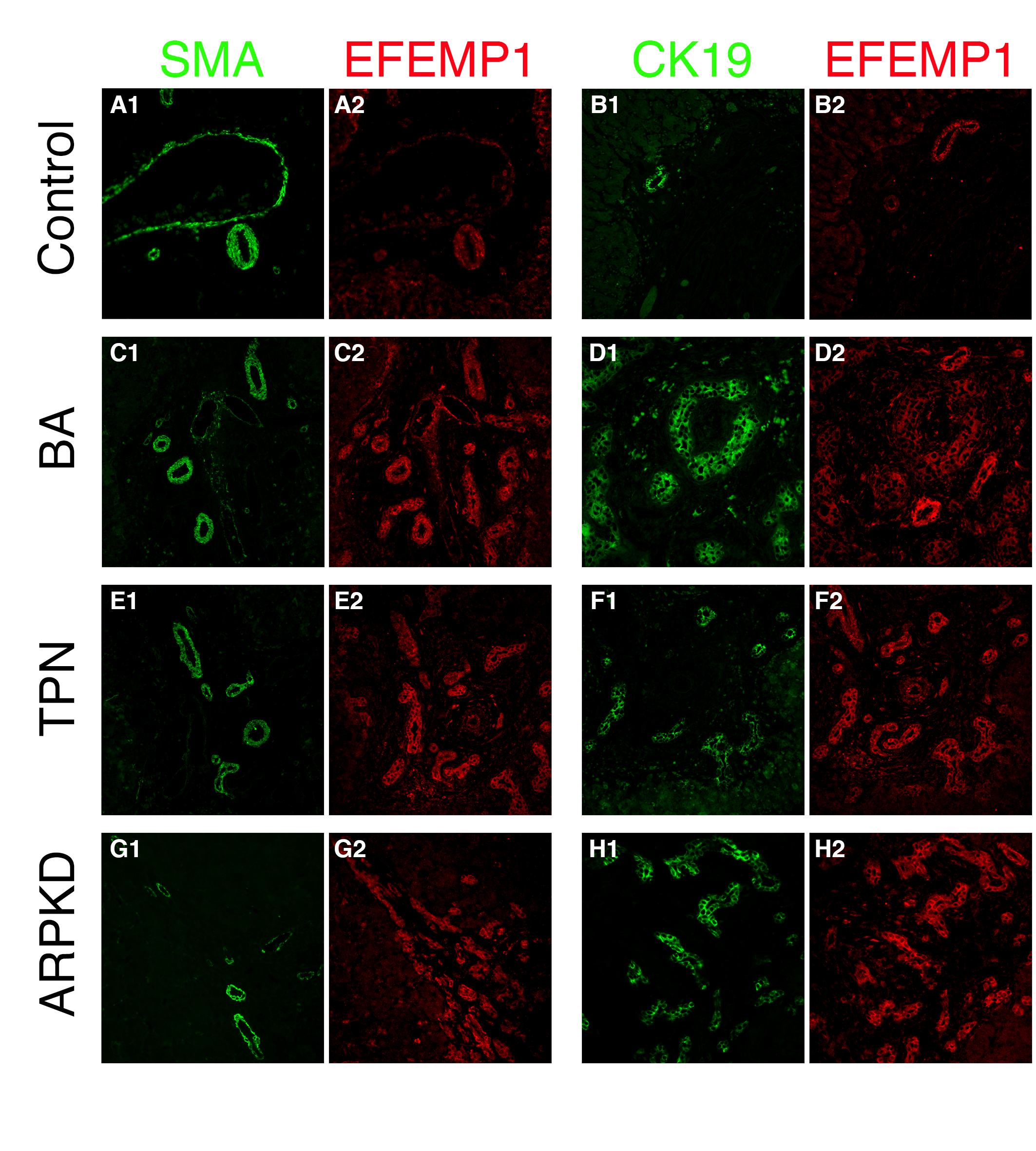

Supplement: S7 Fig — SMA (A1) and EFEMP1 (A2) staining in control tissue; CK19 (B1) and EFEMP1 (B2) staining in control tissue. SMA (C1) and EFEMP1 (C2) staining in BA tissue; CK19 (D1) and EFEMP1 (D2) staining in BA tissue. SMA (E1) and EFEMP1 (E2) staining in TPN tissue; CK19 (F1) and EFEMP1 (F2) staining in TPN tissue. SMA (G1) and EFEMP1 (G2) staining in ARPKD tissue; CK19 (H1) and EFEMP1 (H2) staining in ARPKD tissue. (TIF) [file pgen.1007532.s007.tif]

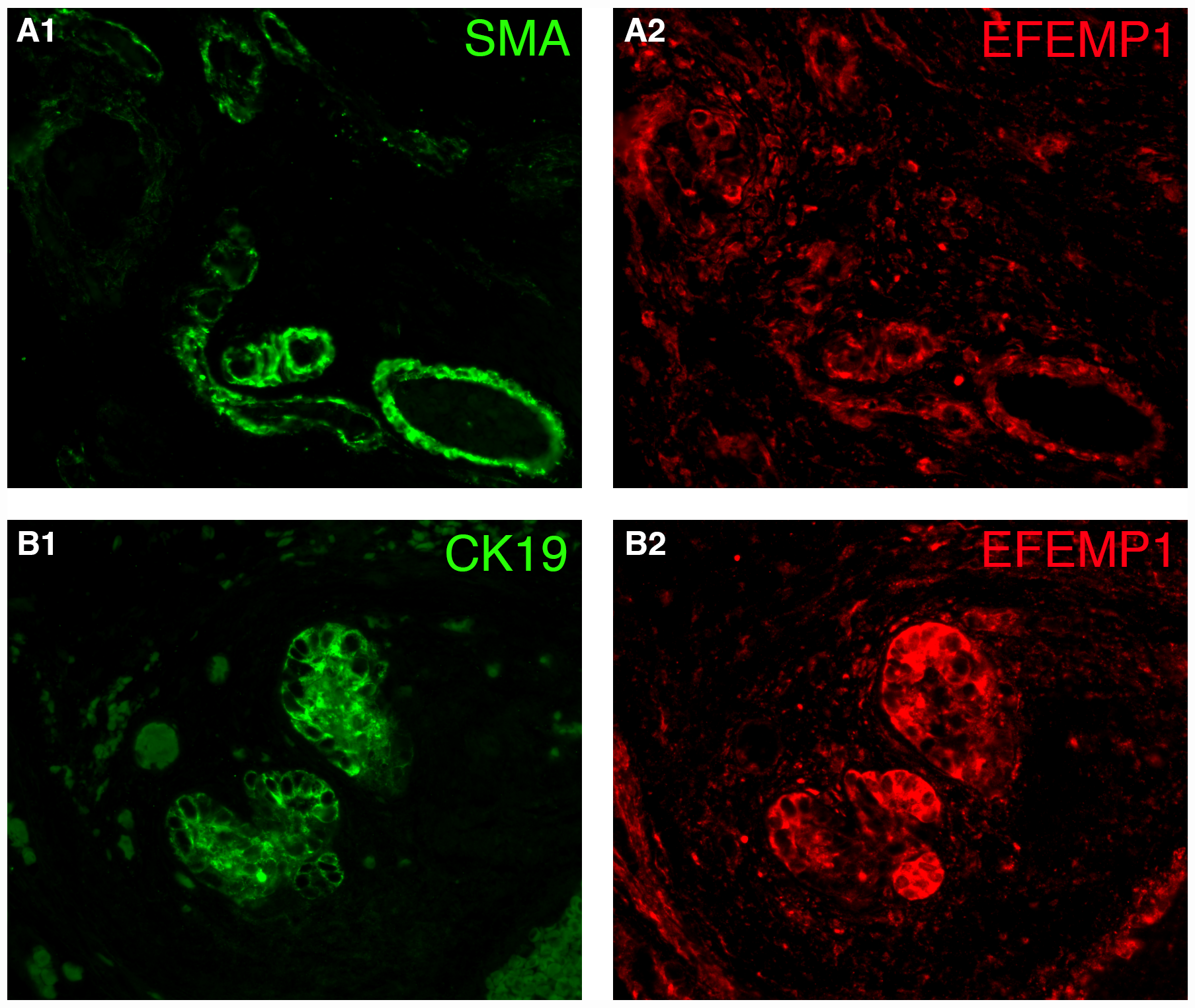

Supplement: S8 Fig — SMA (A1) and EFEMP1 (A2); CK19 (B1) and EFEMP1 (B2) staining in BA tissue. (TIF) [file pgen.1007532.s008.tif]

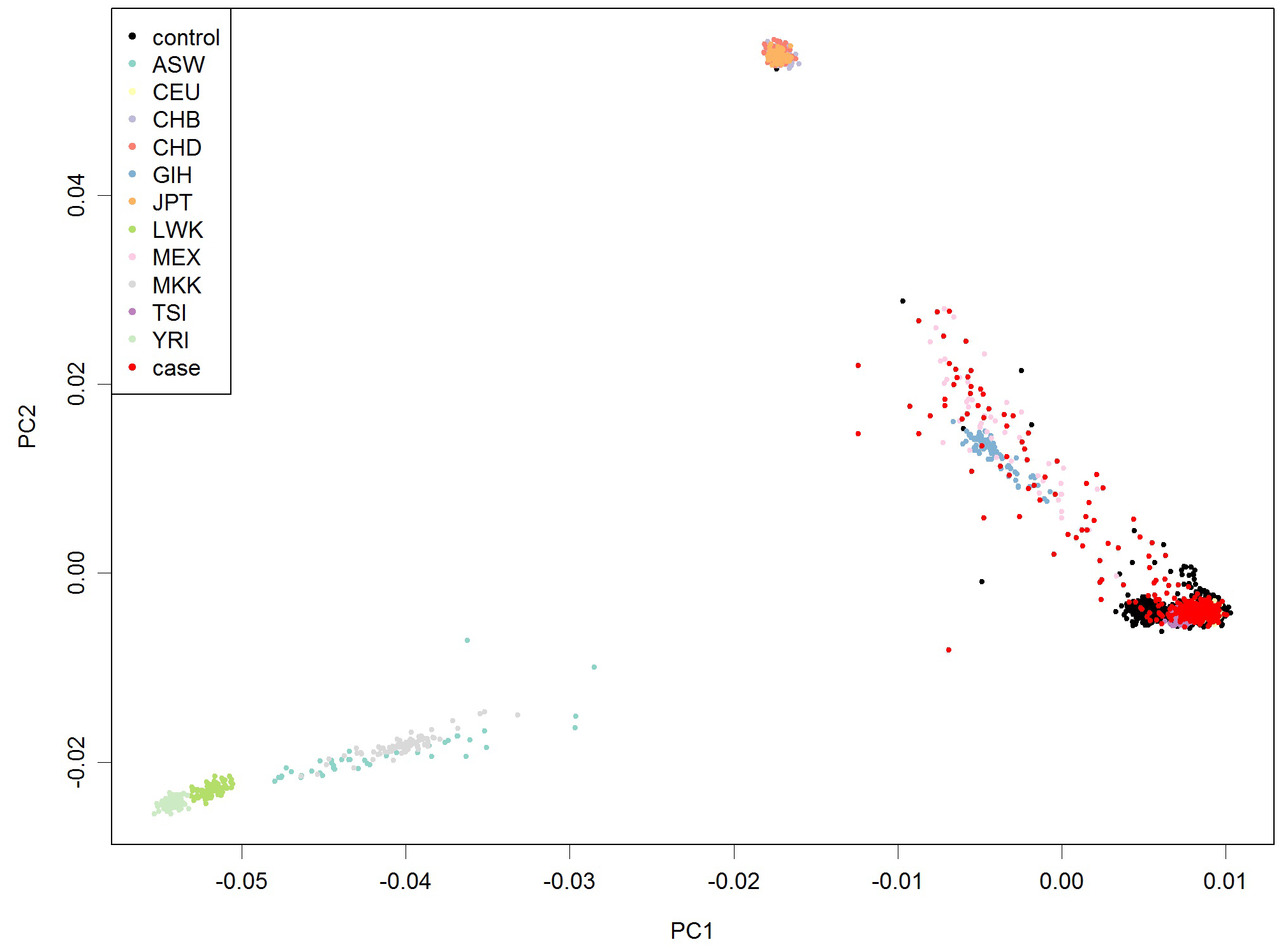

Supplement: S9 Fig — Cases are shown in red and controls are shown in black. (TIF) [file pgen.1007532.s009.tif]

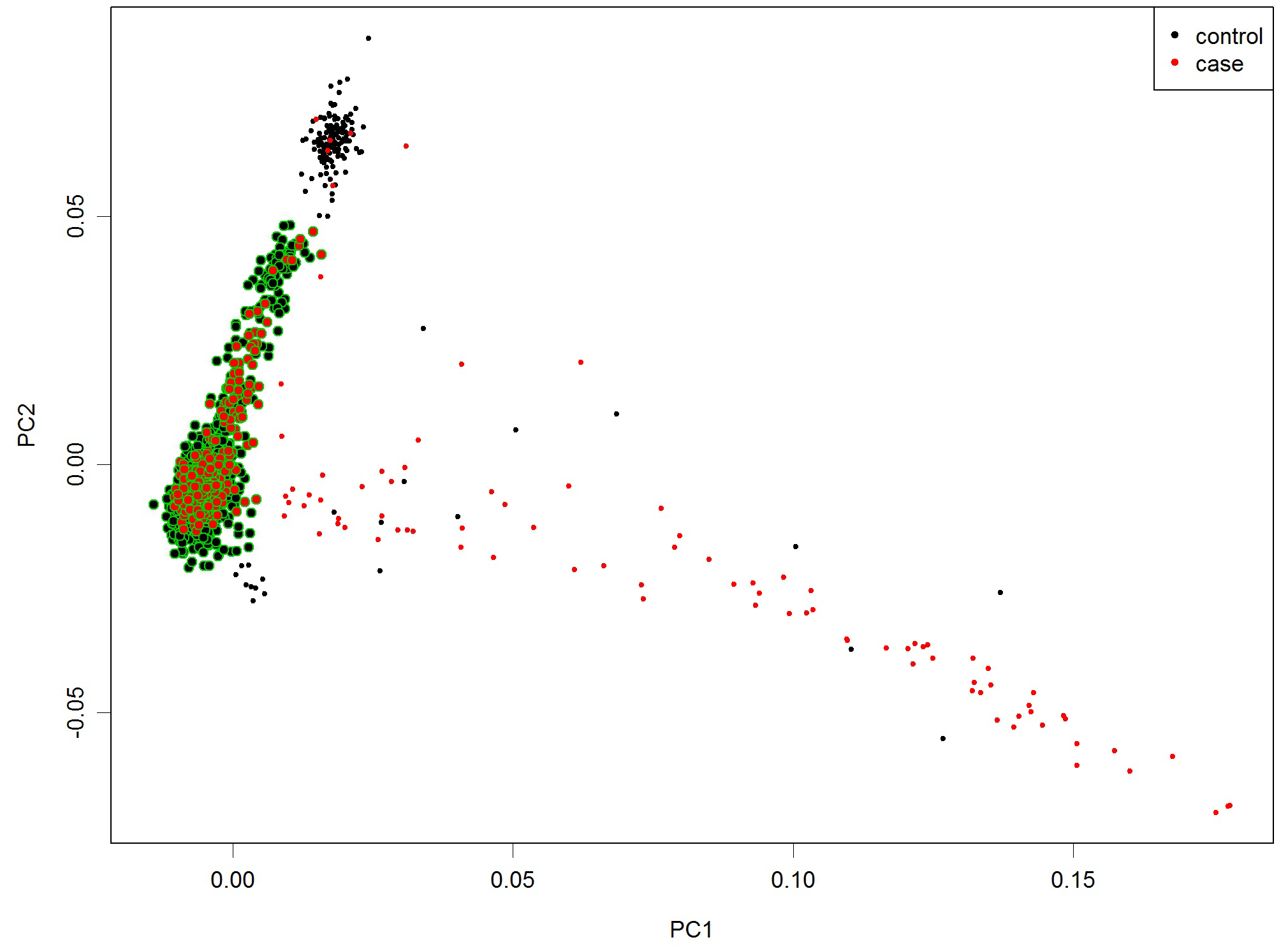

Supplement: S10 Fig — Cases are shown in red and controls are shown in black. The OPTICS clustering algorithm was applied to the first two PCs of 432 BA cases and 1876 AREDS controls. Green circles the selected 343 cases and 1716 controls of European ancestry, which are in one cluster with distance less than 0.003. (TIF) [file pgen.1007532.s010.tif]

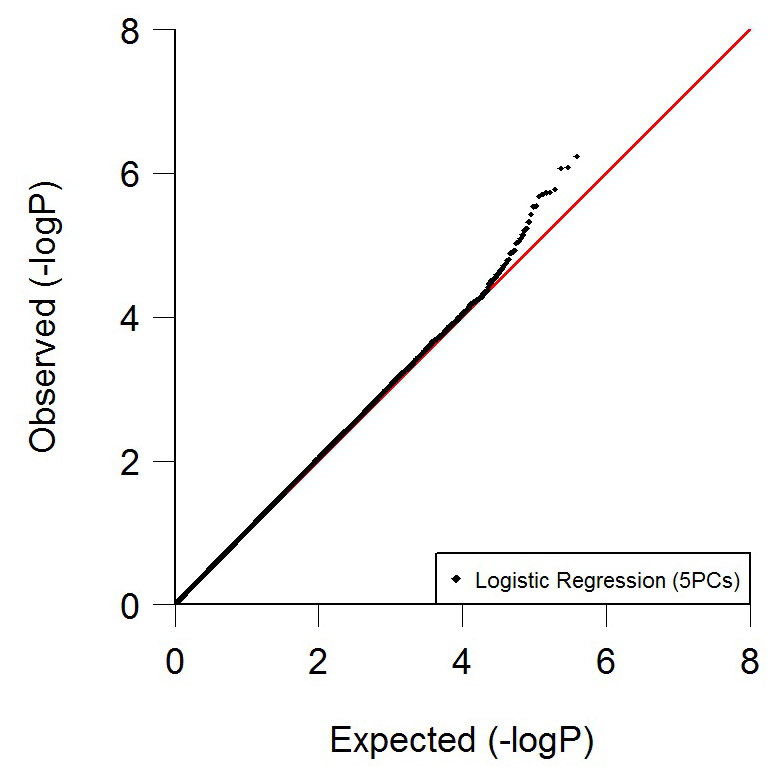

Supplement: S11 Fig — The observed P-values from adjusted logistic regression are plotted against the expected P-values assuming a null hypothesis of no association. No evidence of inflation due to population stratification or other sources of bias was detected. Genomic inflation factor (λ) was estimated as 1. (TIF) [file pgen.1007532.s011.tif]
