# Supplementary material for: A genome-wide association study identifies a susceptibility locus for biliary atresia on 2p16.1 within the gene EFEMP1
Source: PLoS Genet. 2018 Aug 13;14(8):e1007532. doi: 10.1371/journal.pgen.1007532 (PMC6107291; doi:10.1371/journal.pgen.1007532)
Supplement: S1 Table — (DOCX) [file pgen.1007532.s012.docx]

**Table S1.** Genotyped SNPs reaching the suggestive significance threshold (*P* < 1x10^-5^) in the isolated BA cohort. *P*-values were calculated by logistic regression test under additive model adjusted for top five PCs.

|  | | | | | | | |  |  |
| --- | --- | --- | --- | --- | --- | --- | --- | --- | --- |
| **Chromosome** | **SNP** | **Position** | | **Alleles (minor/major)** | **MAF (cases)** | **MAF (controls)** | **Odds ratio**  **(95% CI)** | ***P*-value** | **Nearby genes (kb away)** |
| 1p31.1 | rs356287 | 80977963 | | G/A | 0.46 | 0.36 | 1.46 (1.24, 1.73) | 8.75 × 10^-6^ | *LOC101927412* (23) |
| 1q32.2 | rs12023563 | 210436110 | | A/G | 0.23 | 0.32 | 0.63 (0.52, 0.77) | 3.76 × 10^-6^ | *SERTAD4* (-20) |
| 2p23.2 | rs10432708 | 29534329 | | T/C | 0.37 | 0.46 | 0.67 (0.57, 0.80) | 6.19 × 10^-6^ | *ALK* (0) |
|  | rs4666199 | 29535197 | | C/T | 0.37 | 0.47 | 0.67 (0.56, 0.79) | 4.77 × 10^-6^ | *ALK* (0) |
|  | rs1358516 | 29536711 | | A/G | 0.36 | 0.45 | 0.66 (0.56, 0.79) | 2.93 × 10^-6^ | *ALK* (0) |
| 2p16.1 | rs1346786 | 56108333 | | T/C | 0.39 | 0.29 | 1.57 (1.31, 1.88) | 8.56 × 10^-7^ | *EFEMP1* (0) |
|  | rs11125609 | 56115834 | | C/T | 0.36 | 0.28 | 1.51 (1.26, 1.80) | 8.09 × 10^-6^ | *EFEMP1* (0) |
|  | rs10865291 | 56118046 | | A/G | 0.43 | 0.33 | 1.56 (1.31, 1.86) | 5.85 × 10^-7^ | *EFEMP1* (0) |
|  | rs727878 | 56119657 | | T/C | 0.43 | 0.33 | 1.53 (1.28, 1.82) | 1.85 × 10^-6^ | *EFEMP1* (0) |
|  | rs2868431 | 56119967 | | A/G | 0.44 | 0.34 | 1.51 (1.27, 1.79) | 2.86 × 10^-6^ | *EFEMP1* (0) |
|  | rs80303336 | 56121569 | | T/G | 0.42 | 0.32 | 1.53 (1.29, 1.82) | 1.68 × 10^-6^ | *EFEMP1* (0) |
| 2q37.3 | rs10173589 | 238457684 | | G/A | 0.27 | 0.20 | 1.55 (1.28, 1.87) | 5.81 × 10^-6^ | *MLPH* (0) |
| 3p24.2 | rs74795082 | 24778322 | | T/C | 0.06 | 0.12 | 0.47 (0.34, 0.66) | 9.21 × 10^-6^ | *THRB* (92) |
| 6p22.3 | rs72826622 | 20193704 | | T/C | 0.12 | 0.07 | 1.92 (1.47, 2.50) | 1.87 × 10^-6^ | *MBOAT1* (0) |
|  | rs9460468 | 20194650 | | C/T | 0.12 | 0.07 | 1.96 (1.50, 2.57) | 8.24 × 10^-7^ | *MBOAT1* (0) |
| 6q24.3 | rs17078277 | 148695175 | | G/A | 0.10 | 0.05 | 2.01 (1.54, 2.81) | 1.95 × 10^-6^ | *SASH1* (0) |
|  | rs58833571 | 148701214 | | A/C | 0.10 | 0.05 | 2.01 (1.53, 2.80) | 2.09 × 10^-6^ | *SASH1* (0) |
| 16p13.1 | rs4781487 | 13555023 | | G/A | 0.44 | 0.35 | 1.47 (1.25, 1.75) | 7.16 × 10^-6^ | *SHISA9* (221) |
| 20q13.2 | rs6091375 | 50406630 | | G/T | 0.02 | 0.06 | 0.29 (0.17, 0.50) | 9.51 × 10^-6^ | *SALL4* (0) |
| **MAF**, minor allele frequency. | | |  |  |  |  |  |  |  |
