# Supplementary material for: A genome-wide association study identifies a susceptibility locus for biliary atresia on 2p16.1 within the gene EFEMP1
Source: PLoS Genet. 2018 Aug 13;14(8):e1007532. doi: 10.1371/journal.pgen.1007532 (PMC6107291; doi:10.1371/journal.pgen.1007532)
Supplement: S2 Table — (DOCX) [file pgen.1007532.s013.docx]

**Table S2.** Conditional association test on genotyped SNPs in the *EFEMP1* gene that reached suggestive significance in the isolated BA cohort. *P*-value conditional on rs10865291 and r-square correlations with rs10865291 in cases and controls separately.

|  | | | | | |
| --- | --- | --- | --- | --- | --- |
| **SNP** | **Position** | ***P*-value** | **Conditional *P*-value on rs10865291** | **r^2^ with rs10865291**  **cases controls** |  |
| rs1346786 | 56108333 | 8.56 × 10^-7^ | 0.041 | 0.55 0.52 |  |
| rs11125609 | 56115834 | 8.09 × 10^-6^ | 0.415 | 0.66 0.66 |  |
| rs727878 | 56119657 | 1.85 × 10^-6^ | NA* | 1 1 |  |
| rs2868431 | 56119967 | 2.86 × 10^-6^ | 0.391 | 0.96 0.94 |  |
| rs80303336 | 56121569 | 1.68 × 10^-6^ | 0.742 | 0.93 0.94 |  |
| **NA***, not available because rs727878 is completely correlated with rs10865291 in cases and controls. | | | | |  |
