# Supplementary material for: A genome-wide association study identifies a susceptibility locus for biliary atresia on 2p16.1 within the gene EFEMP1
Source: PLoS Genet. 2018 Aug 13;14(8):e1007532. doi: 10.1371/journal.pgen.1007532 (PMC6107291; doi:10.1371/journal.pgen.1007532)
Supplement: S3 Table — (DOCX) [file pgen.1007532.s014.docx]

| **Table S3.**  List of top 25 genes in gene-based association analysis in the isolated BA cohort. | | | |
| --- | --- | --- | --- |
| Chromosome | Gene | #SNPs | *P*-value |
| 2 | *EFEMP1* | 39 | 3.90E-05 |
| 7 | *IGFBP1* | 3 | 6.60E-05 |
| 3 | *CHST2* | 3 | 0.000144 |
| 16 | *TMED6* | 8 | 0.000323 |
| 11 | *PDE2A* | 58 | 0.000434 |
| 20 | *SALL4* | 15 | 0.000462 |
| 14 | *DTD2* | 4 | 0.000465 |
| 18 | *LOC102724651* | 10 | 0.000583999 |
| 15 | *PYGO1* | 29 | 0.000736999 |
| 10 | *LBX1-AS1* | 5 | 0.000746999 |
| 1 | *MATN1-AS1* | 5 | 0.000899999 |
| 17 | *SUPT4H1* | 3 | 0.00095999 |
| 3 | *CISH* | 2 | 0.00095999 |
| 19 | *LOC284344* | 2 | 0.00101999 |
| 2 | *FAM110C* | 4 | 0.00104999 |
| 19 | *LGI4* | 9 | 0.001099989 |
| 14 | *LOC101927124* | 9 | 0.001109989 |
| 4 | *HADH* | 16 | 0.001109989 |
| 2 | *LOC151174* | 9 | 0.001129989 |
| 9 | *CNTFR-AS1* | 8 | 0.001149989 |
| 17 | *MTMR4* | 5 | 0.001179988 |
| 6 | *HACE1* | 40 | 0.001179988 |
| 4 | *IL15* | 34 | 0.001209988 |
| 11 | *MAP6* | 32 | 0.001289987 |
| 14 | *KTN1-AS1* | 4 | 0.001289987 |
