# Supplementary material for: A genome-wide association study identifies a susceptibility locus for biliary atresia on 2p16.1 within the gene EFEMP1
Source: PLoS Genet. 2018 Aug 13;14(8):e1007532. doi: 10.1371/journal.pgen.1007532 (PMC6107291; doi:10.1371/journal.pgen.1007532)
Supplement: S4 Table — (DOCX) [file pgen.1007532.s015.docx]

**Table S4.** Meta-analysis on 13 genotyped SNPs outside of 2p16.1 reaching *P* < 1 × 10^-5^ in the isolated BA cohort.

|  | | | | | | | | | |  |  | |  | |  | | |  |
| --- | --- | --- | --- | --- | --- | --- | --- | --- | --- | --- | --- | --- | --- | --- | --- | --- | --- | --- |
| **SNP** | **Position** | **Alleles (minor/major)** | **Isolated BA cohort**  **MAF (cases)** | **MAF (controls)** | **Odds ratio**  **(95% CI)** | | | ***P*-value** | | **Non-isolated BA cohort MAF (cases)** | **MAF (controls)** | | **Odds ratio**  **(95% CI)** | | ***P*-value** | | | **Meta-analysis *P*-value** |
| rs356287 | Chr1:80977963 | G/A | 0.46 | 0.36 | 1.48 (1.25, 1.74) | | | 1.20 × 10^-5^ | | 0.41 | 0.36 | | 1.22 (0.90, 1.64) | | 0.109 | | | 4.09 × 10^-6^ |
| rs12023563 | Chr1:210436110 | A/G | 0.23 | 0.32 | 0.63 (0.52, 0.77) | | | 2.89 × 10^-6^ | | 0.27 | 0.32 | | 0.80 (0.58, 1.10) | | 0.285 | | | 3.91 × 10^-6^ |
| rs10432708 | Chr2:29534329 | T/C | 0.37 | 0.46 | 1.46 (1.23, 1.72) | | | 3.91 × 10^-6^ | | 0.43 | 0.42 | | 0.99 (0.74, 1.33) | | 0.567 | | | 1.47× 10^-4^ |
| rs4666199 | Chr2:29535197 | C/T | 0.37 | 0.47 | 1.46 (1.23, 1.72) | | | 3.82 × 10^-6^ | | 0.43 | 0.43 | | 1.00 (0.75, 1.35) | | 0.627 | | | 1.26× 10^-4^ |
| rs1358516 | Chr2:29536711 | A/G | 0.36 | 0.45 | 0.68, (0.57, 0.80) | | | 2.43× 10^-6^ | | 0.42 | 0.42 | | 1.01 (0.75, 1.36) | | 0.579 | | | 9.47 × 10^-5^ |
| rs10173589 | Chr2:238457684 | G/A | 0.27 | 0.20 | 1.49 (1.24, 1.80) | | | 4.65 × 10^-6^ | | 0.21 | 0.21 | | 0.96 (0.67, 1.38) | | 0.697 | | | 1.25× 10^-4^ |
| rs74795082 | Chr3:24778322 | T/C | 0.06 | 0.12 | 0.48 (0.34, 0.66) | | | 5.49× 10^-6^ | | 0.14 | 0.15 | | 0.94 (0.62, 1.42) | | 0.929 | | | 1.01× 10^-4^ |
| rs72826622 | Chr6:20193704 | T/C | 0.12 | 0.07 | 1.96 (1.51, 2.55) | | | 1.28× 10^-6^ | | 0.07 | 0.10 | | 0.67 (0.39, 1.16) | | 0.210 | | | 1.95× 10^-4^ |
| rs9460468 | Chr6:20194650 | C/T | 0.12 | 0.07 | 1.96 (1.51, 2.56) | | | 1.19× 10^-6^ | | 0.07 | 0.10 | | 0.67 (0.39, 1.15) | | 0.203 | | | 1.89× 10^-4^ |
| rs17078277 | Chr6:148695175 | G/A | 0.10 | 0.05 | 2.10 (1.56, 2.83) | | | 9.90× 10^-7^ | | 0.06 | 0.06 | | 1.09 (0.60, 2.01) | | 0.859 | | | 2.54 × 10^-5^ |
| rs58833571 | Chr6:148701214 | A/C | 0.10 | 0.05 | 2.09 (1.55, 2.80) | | | 1.19× 10^-6^ | | 0.07 | 0.06 | | 1.13 (0.62, 2.07) | | 0.962 | | | 2.13 × 10^-5^ |
| rs4781487 | Chr16:13555023 | G/A | 0.44 | 0.35 | 1.47 (1.24, 1.73) | | | 6.54× 10^-6^ | | 0.33 | 0.38 | | 0.83 (0.61, 1.12) | | 0.216 | | | 8.84× 10^-4^ |
| rs6091375 | Chr20:50406630 | G/T | 0.02 | 0.06 | 0.36 (0.22, 0.60) | | | 1.28× 10^-5^ | | 0.08 | 0.07 | | 1.27 (0.73, 2.22) | | 0.378 | | | 8.77× 10^-4^ |
| *P*-values were calculated by SNPTEST using the frequentist likelihood score method under an additive genetic effect model adjusted by statistically significant principal components, and meta-analysis *P*-values were calculated using METAL. | | | | | | | | | | | | | | | | | | |
| MAF: minor allele frequency. CI: confidence interval. | | | | | |  |  | |  | | |  | |  | |  |  | |
