# Supplementary material for: A genome-wide association study identifies a susceptibility locus for biliary atresia on 2p16.1 within the gene EFEMP1
Source: PLoS Genet. 2018 Aug 13;14(8):e1007532. doi: 10.1371/journal.pgen.1007532 (PMC6107291; doi:10.1371/journal.pgen.1007532)
Supplement: S5 Table — (DOCX) [file pgen.1007532.s016.docx]

**Table S5**. Characteristics of 17 human liver specimens used for *EFEMP1* expression analysis.

| Sample Name | Phenotype | Age at collection (years) | Sex |
| --- | --- | --- | --- |
| Control1 | Normal liver adjacent to tumor | 13.82 | Male |
| Control2 | Citrullinemia | 0.94 | Female |
| Control3 | Citrullinemia | 1.37 | Female |
| Control4 | Normal liver adjacent to tumor | unknown | unknown |
| Control5 | Propionic Acidemia | 2.34 | Male |
| BA1 | Biliary atresia | 5.47 | Male |
| BA2 | Biliary atresia | 0.74 | Male |
| BA3 | Biliary atresia | 11.17 | Female |
| BA4 | Biliary atresia | 1.07 | Female |
| BA5 | Biliary atresia | 0.81 | Male |
| Disease Control1 | Primary sclerosing cholangitis | 15.68 | Male |
| Disease Control2 | Cystic fibrosis | 14.32 | Female |
| Disease Control3 | Autoimmune Hepatitis | 12.96 | Male |
| Disease Control4 | Alagille syndrome | 15.17 | Female |
| Disease Control5 | Alagille syndrome | 3.85 | Female |
| Disease Control6 | Alagille syndrome | 13.99 | Male |
| Disease Control7 | Primary sclerosing cholangitis | 3.45 | Male |
|  | | |  |
